# Grid-Scale Impact of Climate Change and Human Influence on Soil Erosion within East African Highlands (Kagera Basin)

**DOI:** 10.3390/ijerph18052775

**Published:** 2021-03-09

**Authors:** Chaodong Li, Zhanbin Li, Mingyi Yang, Bo Ma, Baiqun Wang

**Affiliations:** Institute of Soil and Water Conservation, Northwest A&F University, Yangling 712100, China; licd2020@163.com (C.L.); soilcrop@163.com (B.M.); bqwang@ms.iswc.ac.cn (B.W.)

**Keywords:** climate change, human influence, soil erosion, neighborhood analysis, grey relation analysis

## Abstract

Under global climate change and pressure from human activities, soil erosion is becoming a major concern in the quest for regional sustainable development in the Kagera basin (KB). However, few studies in this region have comprehensively considered the impact of climate change and human influence on soil erosion, and the associated processes are unclear. Based on the premise of quantifying climate change, human influence, and soil erosion, this study undertook a neighborhood analysis as the theoretical support, for a grey relation analysis which was conducted to realize the qualitative assessment of the influence of climate change and human activities on soil erosion. The results show that 90.32% of the KB saw climate change as having a greater influence on soil erosion than human influence, with the remaining area 9.68% seeing human influence having a greater impact than climate change, mainly as a result of the effect of rangeland and farmland. The average soil erosion rate of the KB shows a very low level (10.54 t ha^−1^ yr^−1^), with rangeland and farmland being the main land use/land cover (LULC) types that see soil loss, followed by forest, wetland, and built-up areas. The climate change trends of the KB show the most dramatic changes in the northeast and southwest, gradually decreasing towards the line crossing from the Birunga National Park (Rwanda) to the Keza district (Tanzania). The human influence intensity (HII) shows a high level in the KB (21.93), where it is higher in the west and lower in the east of the basin.

## 1. Introduction

Soil erosion has become a major challenge for global sustainable development [1], and the impact of climate change and human influence on it has been well established worldwide [2,3,4,5]. As one of the sources of the Nile River, the Kagera Basin (KB), East Africa, and its ecosystem is related to the livelihoods of the upper reaches of the river, and even the entire Nile River Basin, including regulating hydrological cycles, soil erosion control, and food supply [6]. However, under the pressure of global climate change, increasing population pressure, and the rapid development of the social economy, soil erosion in the basin has become an increasingly serious issue [7].

Soil erosion is a natural process affected by many factors; the main drivers differing between environments. At high latitudes/high altitudes, soil erosion is not only affected by water, but also by wind and freezing/thawing processes [8], with their combined impact affecting regions such as northern China, the Inner Mongolia plateau, northern Europe, and northern Canada. Soil erosion at lower latitudes is mainly driven by water, along with part of the gravitational erosion [8], affecting regions such as southern China, Southeast Asia, and South America. Meteorological factors (precipitation and temperature, etc.) also have an important impact on soil erosion, while human surface activities, such as farm management practices, do not have a negligible impact [9]. All of these factors lead to the formation of a variety of different kinds of erosion distributed across the globe, depending upon the relative importance of the joint action of the various driving forces, meteorological conditions, and surface activities [10]. It is generally believed that climate change affects soil erosion in both direct and indirect ways. The direct impacts are mainly realized through precipitation, in terms of the amount of precipitation, the intensity, and its temporal and spatial distribution [2,10], while the indirect impacts are mainly caused by the joint action of meteorological factors such as air temperature and wind speed, which influences vegetation, soil moisture content, and soil microorganisms [10,11,12]. Hence, it may be said that climate change has both positive and negative effects on soil erosion.

The impact of human activities on soil erosion is more direct than climate change. Human-driven land use/land cover (LULC) changes are considered to be the main cause of soil loss in the dryland basins of sub-Saharan Africa [13]. The absence of comprehensive land development plans may also contribute to accelerating soil erosion, which is particularly acute under the dual pressures of population growth and poverty [1]. As is well known, the extent and type of vegetation coverage are some of the main factors affecting soil erosion levels. The extensive reclamation of slope cropland not only causes the destruction of the original vegetation, but it also changes the soil structure on the surface and accelerates soil erosion [14]. A large number of eucalyptus forests have been felled for use as residential fuel, which has also accelerated this process [15]. Recently, due to the sustained rapid economic development in East Africa, a large amount of infrastructure is being developed, which also contributes to the human influences that affect the natural processes of soil erosion [16].

Understanding the impact of climate change is crucial for predicting future changes in soil erosion and the needs of land management. Previous studies have been mostly based on General Circulation Models (GCMs), which generate climate data with spatial attributes according to representative concentration pathway (RCP) scenarios [17,18]. On this basis, soil erosion models are applied to simulate and predict future soil erosion. Compared with climate change, the intervention of human activity on soil erosion is more intuitive, such as the effects of agriculture [19], changes in LULC [20], and the construction of water conservancy and soil conservation measures. Current research is mostly about the impact of the improvement of farming methods, changes in LULC types, and the arrangement of soil and water conservation measures dealing with soil erosion under individual or overlapping effects [21]. There are also studies based on historical monitoring data, using statistical analysis methods to determine the contribution of climate change and human influence on the runoff and sediment changes at the outlet of the basin [22]. Previous studies have spanned the past and the future while considering spatial scales from basins (regions) to the world as a whole. Most of these works have dealt with only one or more independent factors (LULC, farming methods, agricultural management, and future climate scenario) with regards to the impact of climate change or human influence on soil erosion. However, there is no consensus on the relative importance of the impact of climate change and human influence on levels of soil erosion. Thus, it is urgent to clarify the respective impact of these groups (climate change and human activities) of processes on soil erosion [23]. Therefore, qualitative assessment of the effects and importance of climate change and human influences is presented in this paper, based on neighborhood analysis theory, while adopting the method of grey relation analysis (GRA) by gridding maps of the climate change, human influence, and soil erosion experienced by the basin, to identify how the different factors (climate change and human influence) impact upon soil erosion for different areas. First, soil erosion, human influence intensity, and climate change trends in the KB were assessed separately. Based on these results, GRA of soil erosion, human influence intensity and climate change trends was carried out. This approach can be characterized from the grid-scale analysis of the impact of climate change and human activities on soil erosion, so as to achieve a more accurate policy of regional planning and to develop information management.

## 2. Materials and Methods

### 2.1. Study Area

**Figure 1:**
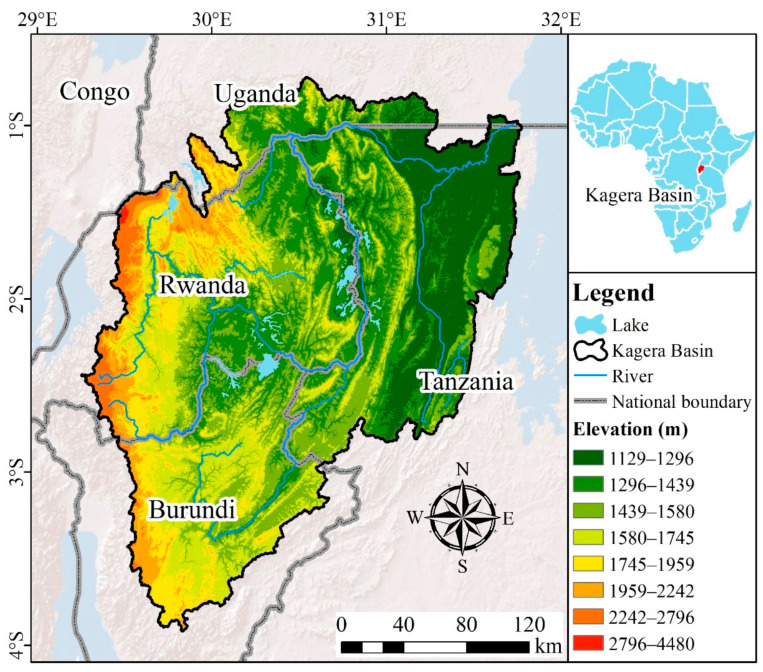
Location of the study area.

The Kagera River (0°45′–3°35′ S, 29°15′–30°51′ E) is located on the East Africa Plateau, the most remote headstream of the Nile River and the largest of the 23 rivers that drain into Lake Victoria, supplying 34% of the annual inflow into the lake. Sediment carried by runoff from the Kagera River is a major part of Lake Victoria’s sediments [24,25]. The KB (Figure 1) covers extends over four countries: Burundi (23% of the basin), Rwanda (34%), Tanzania (35%), and Uganda (8%), with a total area of 60,000 km^2^. Within the basin lies 75% of the land area of Rwanda and 52% of Burundi. The topography of the KB is dominated by mountains and hills, with an altitude range of 1129–4480 m. The average annual temperature in the upper reaches is 18 °C, and for the lower reaches, it is 21 °C. The annual precipitation shows a bimodal trend caused by the double rainy season, namely, March to June and October to December, with a dry season between. The average annual rainfall varies greatly between the upper and lower reaches of the basin, ranging from 800 mm to 2000 mm, respectively, while during the rainy season rainstorms frequently occur.

### 2.2. Overview of the Employed Methods

**Figure 2:**
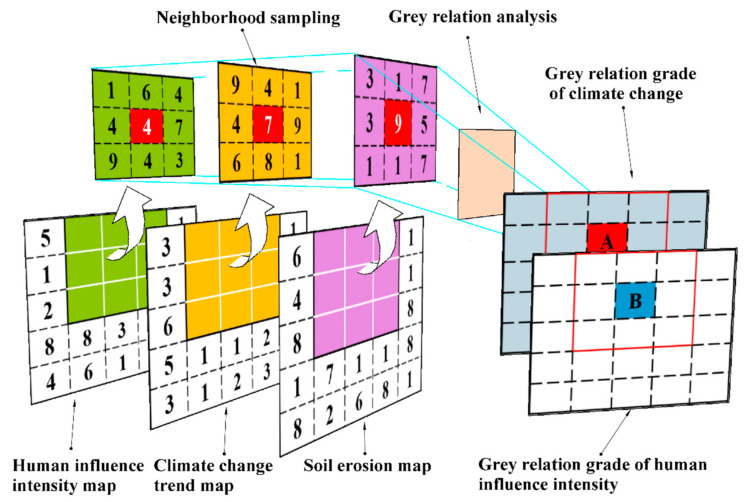
Schematic diagram of the neighborhood sampling for the grey relation analysis (GRA).

The research undertaken for this work is based on neighborhood analysis, which is a type of window analysis. It takes the raster pixel to be calculated as the center and extends to a certain range to the surroundings, and then performs the GRA based on the values of these expanded raster pixels and the central pixel (analysis window) to obtain the grey relation grade of the pixel to be calculated. A 3 × 3 (grid) is selected as the analysis window size. After sampling the soil erosion, climate change, and human influence maps on a grid-by-grid basis, the GRA was carried out to obtain the grey relation grade of the influence of climate change and human activities on soil erosion, and to perform statistical analyses (Figure 2).

**Figure 3:**
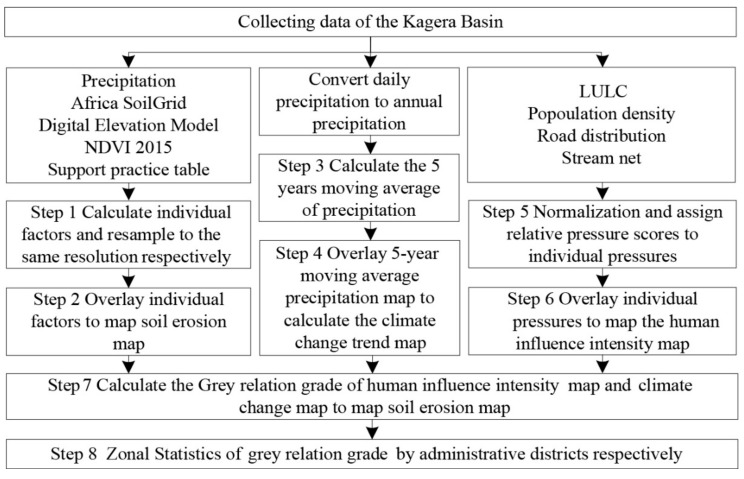
Flowchart depicting the analysis process used in this study. See the text for details about the datasets employed.

As the most widely used model of soil erosion, the Revised Universal Soil Loss Equation (RUSLE) has been successfully applied in East Africa. The nearest neighbor interpolation resampling method was employed to provide the same spatial resolution (500 m) for each soil erosion impacting factor (Figure 3, Step 1). Then, we superimpose the individual factors to obtain the soil erosion map of the KB (Figure 3, Step 2).

Precipitation was selected as the climate change factor that impacts upon soil erosion, and Sen’s Slope Estimator and the Mann–Kendall non-parametric test were used to quantify the climate change factor. Sen’s Slope Estimator is used to quantify the change trend of precipitation, and the Mann–Kendall non-parametric test can quantitatively reflect the significance of the change trend. The precipitation over the KB from 1981 to 2015 was processed as follows. (1) the daily precipitation was combined to give annual precipitation; (2) a moving-point average was used to process the precipitation from 1981 to 2015 for each grid cell, with 5 years as the sliding window (Figure 3, Step 3); (3) Sen’s slope value map was calculated and the Mann–Kendall test was performed, resampling the Sen’s slope value map to 500 m resolution (Figure 3, Step 4).

The human influence map uses the method developed by Sanderson et al. [26], which is based on the comprehensive consideration of the biological, physical, and cultural characteristics of the study area. This study focuses on defining the human influence intensity through geographic indicators, such as LULC, population density, and accessibility (road distribution and stream network) (Figure 3, Step 7). The national scale (AMD0) and districts scale (AMD1) grey relation grades were averaged across the research area (Figure 3, Step 8).

### 2.3. Data Sources and Calculating Method

#### 2.3.1. Data Sources

The present study estimated the long-term daily precipitation for 35 years (1981–2015) using daily precipitation provided by the Climate Hazards Group InfraRed Precipitation with Station data (CHIRPS), which has been used in the study of hydrological forecasts and trend analyses in East Africa (Ethiopia) [27].The soil data are from the Africa Soil Information Service (AfSIS) with a spatial resolution of 250 m [28]. The soil data were used to give the soil texture (percentage of sand, silt, and clay) and soil organic carbon content to obtain soil erodibility. The digital elevation model (DEM), Normalized Difference Vegetation Index (NDVI), and LULC data were downloaded from the United States Geological Survey (USGS) EarthExplorer database. The DEM has a 30-m spatial resolution and was selected to derive the topographic factors. The NDVI used in this study is the MODIS MYD13A1 product [29] with a temporal resolution of 16 days. The images have a spatial resolution of 500 m and are retrieved from daily, atmospherically corrected surface reflectance observations. For this study, annual NDVI is used to develop the cover management factor of the RUSLE, which was obtained using the maximum value composite method. The MODIS MCD12Q1 [30] products were used to extract the LULC. The LULC adopted the International Geosphere-Biosphere Programme (IGBP) classification system [31], and the detailed classification is shown in Table 1. The temporal and spatial resolution of the LULC image are 1 year and 500 m, respectively. Population density is derived from the Africa Continental Population Datasets Version 2.0 [32] published by Worldpop. The spatial resolution of the population data image is 0.0083° (about 1 km at the equator).The Road data for Rwanda, Burundi, Tanzania, and Uganda were downloaded from the African Development Bank Group (valid for the year 2016). Because the roads in the study area are managed by four countries, the road classification is inconsistent, so the data could not be merged smoothly. Therefore, the road surface conditions (paved or unpaved) were selected as the merged standard to obtain the road distribution within the KB.Stream networks were obtained from the LakeVicFish Dataverse [33] https://dataverse.harvard.edu/dataverse/LakeVicFish (accessed on 20 October 2020). The stream networks are not considered to serve as transportation channels, so the classification of the rivers is not carried out, with all channels being treated as the same grade.

#### 2.3.2. Development of the RUSLE Model

The RUSLE has been used worldwide since it was first proposed [34]. As a quantitative model of soil erosion, RUSLE has many advantages, such as convenient data acquisition, simple factor calculation, and so forth. The RUSLE model calculates the average annual soil loss as follows (Equation (1)):

A = R · K · L · S · C · P(1)

where A is average soil loss per unit area per year (t ha^−1^ y^−1^), R is the rainfall erosivity factor (MJ mm ha^−1^ y^−1^), K is the soil erodibility factor (t ha^−1^ h^−1^ ha MJ mm), L is the slope length factor, S is the slope steepness factor, C is the cover and management factor, and P is the support practice factor.

R is a dynamic factor that characterizes the erosivity energy of soil erosion. A method to calculate R for tropical areas based on monthly rainfall developed by [35] is adopted in this study. R is determined by the amount of soil loss caused by the per-unit area rainfall erosivity on the standard plot. K is an attribute of the soil which is related to soil properties and is found using the equation from the Erosion Productivity Impact Calculator (EPIC) [36]. Note, the constant 0.1317 value is used to convert the K factor from the American system to the International System of Units (SI) [37]. Topographic factors were characterized by L and S, which are quantitative descriptions of the terrain. L is influenced by the ratio of rill erosion to inter-rill erosion. S reflects the effect of slope on erosion sediment yield, where the greater the slope, the more severe is the erosion. The formula established by [38] was used for calculating S. C is an important index of the anti-erosion capacity of vegetation cover and refers to the ratio of the amount of soil loss on land covered by vegetation or field management to the soil loss on uncovered bare land under the same underlying surface and rainfall dynamic conditions. We adopted the equation developed by Van Leeuwen and Sammons [39] and revised by Van der Knijff et al., [40]. P reflects the impact of support practices on the annual erosion rate. The closer P is to 0, the lower the soil erosion, which also means that the soil erosion prevention practices are more effective, and vice versa. The resolution of the data in this study is low (500 m), while fanya-juu terrace, soil contour bund, and contour tillage are the main support practices of the KB, which cannot be effectively expressed at such a resolution, with fanya-juu terrace being the most extensive support practice in the KB [41]. Therefore, we take 1 as the support practice factor in this study.

#### 2.3.3. Climate Change Trend Analyses

Parametric and non-parametric tests are two commonly used long-term weather data test methods. Parametric trend testing requires data to be independent and normally distributed, while non-parametric trend testing requires only independent data. Two non-parametric methods (Sen’s slope estimator and the Mann–Kendall test) were used to detect the climate change trend over the KB for each grid point. Sen’s slope estimator [42] is used to calculate trends in climate change while the Mann–Kendall test [43,44] provides a measure (Z_S_) that indicates whether the long-term change of a variable is significant or not. If the absolute value of Z_S_ is greater than 1.96 or 2.58, it means that the trend has passed the 95% and 99% significance level tests, respectively.

#### 2.3.4. Mapping the Human Influence Intensity

The human influence intensity (HII) assessment method proposed by Sanderson et al., [26] can be used over a global scales. In the KB, due to the frequently shifted and scattered LULC, high population density, and the distribution characteristics of the road and stream networks, these factors were selected as the individual pressure factors to map the HII in 2015.

##### Land Use/Land Cover (LULC)

Land transformation is the most direct manifestation of human influence on the environment [45]. With the support of relevant local literature [46,47], combined with previous studies [26,48], we assign different scores for each land use type, as shown in Table 1.

##### Population Density

Human influence on the environment is proportional to population density [49,50], but the carrying capacity of the natural environment is limited, so population density has a threshold in term of its impact on the environment. This means human influence on the environment will reach a maximum when the population density reaches a certain value. Cardillo et al. [51] experimentally showed that the human impact on the environment will stabilize after the population density reaches 50 inhabitants/km^2^. Based on the above values, considering that the population density of Africa is higher than the world population density [52], in this study we assigned scores to the population density in each grid, where scores for population density in the range of 0–50 inhabitants/km^2^ increased is linearly from 0 to 10. We assigned all population densities greater than 50 inhabitants/km^2^ a score of 10.

##### Accessibility

Accessibility factors generally consider road distribution, stream networks, and coastlines as the indicators of the environment which are correlated with human influence [36]. However, the study area is located in the interior of East Africa, so coastlines are not considered, with only roads and stream networks being used. We consider the extensive development in road construction [14], although the channels are not used for transportation, but rather for the protection of the residents’ domestic water supplies [53]. According to relevant local research [54,55], the maximum impact distance of the roads and stream networks are set to 15 km and 5 km, respectively, and relevant values have been assigned to these and intermediate distances (Table 2).

#### 2.3.5. Grey Relation Analysis (GRA)

The GRA is a multi-factor statistical analysis method calling upon the grey system theory developed by Deng [56]. The GRA can determine the grade of correlation between factors based on the similarity of the geometric shapes of the change curves of various factors. The detailed steps are as follows: Step 1: Normalize the original data. Step 2: Reference sequence definition. Step 3: Grey relation coefficient calculation. Step 4: Grey relation grade calculation. In this study, the formula developed by Winarni et al. [57] was used to obtain the Grey relation grade.

## 3. Results

### 3.1. Soil Erosion in the Kagera Basin

**Figure 4:**
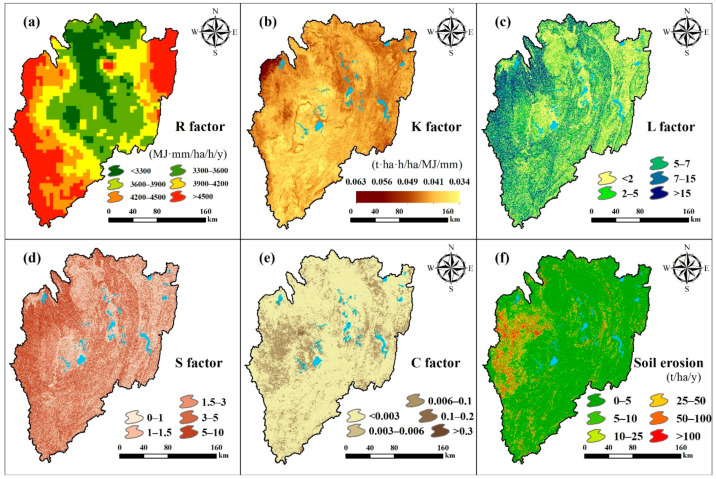
Maps of the Revised Universal Soil Loss Equation (RUSLE) factors for the Kagera basin (KB); (**a**) rainfall erosivity factor; (**b**) soil erodibility factor; (**c**) slope length factor, (**d**) slope factor; (**e**) cover management; (**f**) soil erosion.

After calculating each factor of the RUSLE separately (Figure 4a–e), the simulation results show that the KB’s average annual soil erosion rate in 2015 was 10.54 t ha^−1^ yr^−1^, and the total annual soil loss was 60.17 million tons. According to the classification of soil erosion severity [58], it may be classified into 6 classes as shown in Table 3. The soil erosion of the KB is dominated by very low (75.14%) levels, followed by high (8.16%), low (7.42%), very high (4.41%), severe (2.75%), and moderate (2.12%) levels. The spatial distribution of the soil erosion rates is shown in Figure 4f, where high, very high, and severe soil erosion occurs in the western region of the KB, with moderate soil erosion predominate in the central and eastern regions. Overlaying the soil erosion map with the LULC reveals the erosion intensity and amount of erosion of different LULC types. In this study, land cover types were reclassified into seven main categories for statistical analysis (Table 4). As shown in Table 5, rangeland and farmland are the main LULC types, with areas of 35,071 km^2^, and 20,330 km^2^, respectively, accounting for 61.35% and 35.56% of the total area, respectively, and the amount of soil loss from them accounted for 49.60% and 45.97% of the total for the KB, respectively. For the forest, wetland, and built-up areas, which cover 3.09% of the basin’s area, the amount of soil loss accounts for 4.43% of the total of the basin. The average soil erosion rate of the different LULC types is 33.406 t ha^−1^ yr^−1^, with forest showing 0.71 t ha^−1^ yr^−1^, rangeland 8.51 t ha^−1^ yr^−1^, wetland 11.77 t ha^−1^ yr^−1^, farmland 13.61 t ha^−1^ yr^−1^, and built-up areas 132.43 t ha^−1^ yr^−1^.

Generally, the soil erosion of the KB is classified as being very low. High, very high, and severe erosion mainly occurs in Rwanda in the west of the KB. Farmland is the main area of serious soil erosion in the KB, while the erosion in the central and eastern parts mainly occurred in rangeland. Although the soil erosion rate of rangeland areas is lower than that of farmland, it also results in considerable soil loss due to its large area. Finally, while soil loss caused by urban expansion is very small, it cannot be ignored due to the resulting high soil erosion rates.

### 3.2. Climate Change Trends in the KB

**Figure 5:**
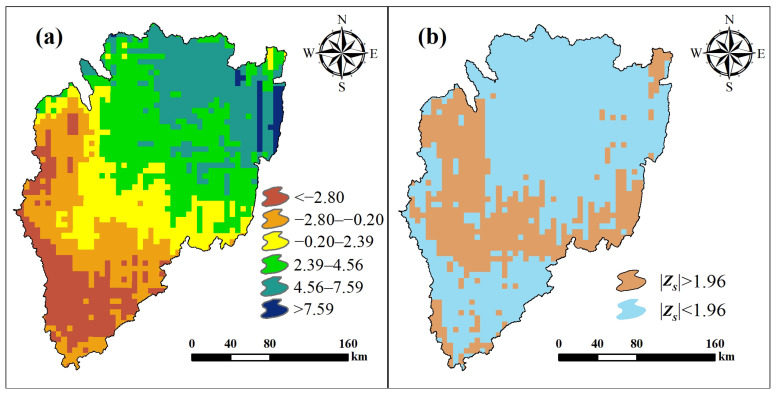
Trend analysis of precipitation; (**a**) Sen’s Slope value map; (**b**) the MannKendall test value map.

Figure 5a depicts the spatial distribution of the rainfall trends between 1981 and 2015 for the KB. The results of the Mann–Kendall test for annual precipitation over this period are shown in Figure 5b, where 66.68% of the grids passed the significance tests (i.e., |Z_s_| > 1.96).

The Sen’s Slope value map of the KB presents a decreasing trend from northeast to southwest. The maximum value of 13.13 appears at Maruku-Katoma in the northeast, and the minimum value of 8.95 appears in the Nyungwe National Park in the southwest. The map appears to be divided into two parts (the southwest part and northeast part) by a line from the Birunga National Park (Rwanda) to the Keza district (Tanzania). In the southwest, Sen’s Slope value is less than 0 and rainfall presents a significant decreasing trend (Z_s_ < −1.96). Sen’s Slope value in the northeast of the KB is greater than 0, and the rainfall shows a significant increasing trend (Z_s_ > 1.96).

### 3.3. Human Influence Intensity in the KB

**Figure 6:**
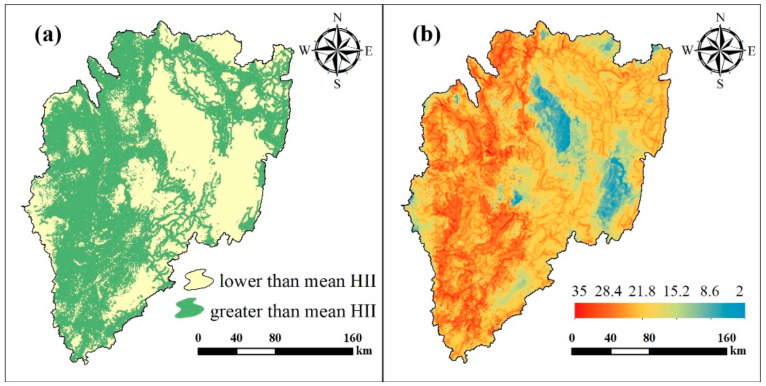
Human influence intensity (HII) map of the KB showing: (**a**) HII divided by its mean value; (**b**) the HII map of the KB.

The mean HII value over the KB as found by this study was 21.93, while the maximum grid value was 32, which indicates that the HII was generally high in the KB for 2015. In addition, the percentage of grid points with HII values lower than the average HII value was 42.21%, and 57.79% were higher than the average (Figure 6a). It can be seen that the western region of the KB (west of the Akagera National Park) is relatively high, where the natural conditions of this region are more suitable for human activities; thus, there is a higher HII value. For other regions, the HII values were lower, especially in the southeastern region of the KB, the central region, and the Nyungwe National Park in the western-most part of the basin. The HII is an index affected by multiple factors and because each independent factor does not involve factor weights in the merging process, the spatial distribution of HII will show the same trend as independent factors within a certain range. For example, the Akagera National Park and the Burigi Game Reserve are rarely considered to be disturbed because they are designated as national natural protected areas (Figure 6b). Population density and road distribution, etc. all show a lower level in this area, hence this is where the lowest HII values in the basin appear.

### 3.4. Impact of Climate Change and Human Influence on Soil Erosion

**Figure 7:**
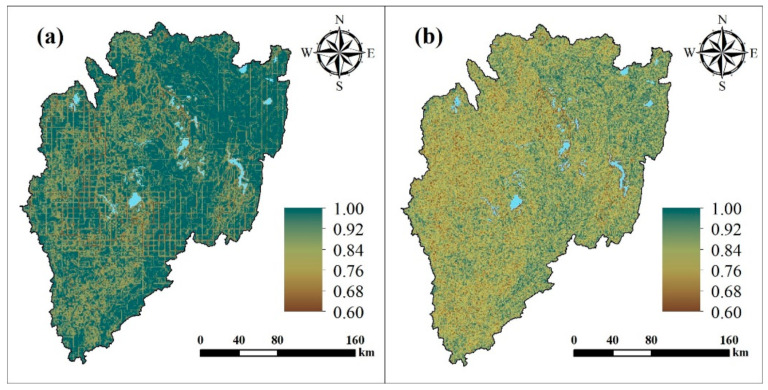
Grey relation grade map of (**a**) climate change and (**b**) human influence.

Figure 7 shows the spatial distribution of the grey relation grade between climate change and human influence on soil erosion, with average values of 0.97 and 0.84, respectively, across the basin. Among them, for 90.32% of the grids, the effect of climate change was greater than that of human influence, and 9.68% of the grids show human influence was greater than climate change.

**Figure 8:**
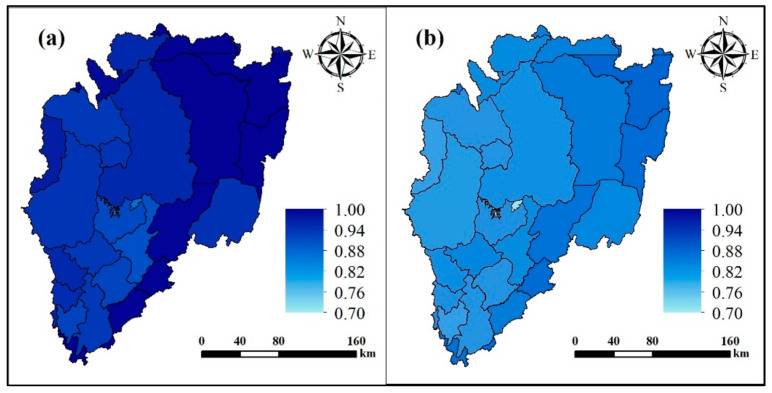
Grey relation grade maps of (**a**) climate change and (**b**) human influence for the AMD1 subdivisions.

The grey relation grade is calculated according to the AMD0 and AMD1 scales. Over the AMD0 scale, the grey relation grade of climate change for the four countries that make up the basin decreases, with values of 0.99, 0.98, 0.94, and 0.94 for Tanzania, Uganda, Rwanda, and Burundi, respectively. The grey relation grade of human influence also shows a decreasing trend of 0.86, 0.83, 0.83, and 0.81 for Tanzania, Uganda, Burundi, and Rwanda, which are 0.86, 0.83, 0.83, and 0.81, respectively. In terms of their spatial distribution, both factors appear to decrease from the northeast to the southwest. Over the AMD1 scale (Figure 8), the change trends are the same as for AMD0 scale, where the grey relation grade for climate change has the largest (0.88) and the smallest (0.80) in Bukoba Rural (Tanzania) and Mwaro (Burundi), respectively. The maximum (0.99) and minimum (0.90) of the grey relation grade for human influence appeared in Ngara (Tanzania) and Muyinga (Burundi), respectively.

## 4. Discussion

### 4.1. Major Factors Influencing Soil Erosion

The soil loss across the KB mainly arises from rangeland and farmland areas, which cover 55,402.25 km^2^, accounting for 96.91% of the total area of the basin. Table 5 shows that 35.56% of the farmland area leads to 45.97% of the total soil loss, mainly due to the reclamation of slope farmland in the west of the KB, which is consistent with the results of [59]. A large area of natural forests and rangeland in Rwanda have been converted into cultivated land [60], which has led to changes in surface vegetation. Rainfall in Rwanda is high and concentrated during the rainy season, and most single-season crops (potatoes, corn, and legumes, etc.) are planted at the beginning of the rainy season, resulting in rainfall that hits the nearly bare surface directly, causing a large amount of soil erosion [14]. However, banana plantations are the main source of food crops in the region, where soil management measures show that these areas directly affect the soil erosion in the area. Meanwhile, studies have shown that appropriate management measures can improve soil health [9], thereby making the soil sustainable.

Under the coercion of high population pressure, a large number of barren slopes were continuously cultivated as slope farmland [14]. Although expanding the area of sloping farmland can alleviate temporary food shortages, it is not sustainable. The expansion of the slope cropland has caused serious soil erosion because of the heavy precipitation in the KB and the steep slopes of the cropland (where the average slope is 13°). Although the built-up LULC types listed in Table 5 saw only 2.48% of soil loss, the soil erosion rate was indeed as high as 132.43 t ha^−1^ yr^−1^. Furthermore, the urbanization of the KB is expanding year by year [61], causing serious damage to the natural environment and vegetation of the surface soil of the built-up lands [62]. Furthermore, the development of infrastructure projects lacks effective soil and water conservation management measures, again causing serious soil erosion [16].

### 4.2. Previous Studies

Through the GRA outlined in Section 3.4, it is found that the impact of climate change on soil erosion is greater than human influence. The results obtained from this work are consistent with Zuo et al. [63], which showed that the impact of climate change on runoff is greater than that of human influence (53.7% > 25.3%), and the impact of climate change on sedimentation is also greater than that of human influence (LULC) (81.0% > 40.6%). However, 9.68% of the area shows that human influence has a greater impact on soil erosion compared to climate change in the KB. To further clarify the composition of the soil erosion area dominated by human influence, the area where human influence was greater than climate change was extracted and cross-analyzed with the LULC, where it was found that the rangeland, farmland, forest, wetland, and built-up areas accounted for 48.79%, 47.72%, 1.67%, 1.39%, and 0.44%, respectively, of the soil erosion. Soil erosion dominated by human influence mainly occurs in the rangeland and farmland areas, followed by forest, wetland, and built-up. Rangeland and farmland are areas that are dominated by human influence, and it is reasonable that the impact of human influence on the soil erosion of such areas is greater than that due to climate change, as verified by Cai [22].The impact of climate change on soil erosion is large-scale and spatially continuous, while the impact of human influence on soil erosion will be affected by the LULC, which is spatially heterogeneous and irregular, although the effect in these parts is more long-term [64,65,66,67].

### 4.3. Uncertainty Analysis

The results in this work involve uncertainties, which not only need to be understood by environmental protection planners, but also by researchers who are interested in improving upon current studies of the KB for future research. During the calculation of the soil erosion map, due to the limitation of the data resolution, the value of the P factor was set to 1, meaning that no soil and water conservation (SWC) measures have been deployed. However, SWC measures have been employed in East Africa [68,69]. For example, Uganda has built bench terraces in its southern region, which were equipped with agroforestry systems [70]. Rwanda began its development of SWC measures, such as terraces, in the 20th century [71]. As of 2013, such measures (progressive terraces and bench terraces) have been implemented over more than 855,114 ha [72]. Tanzania and Burundi have also implemented SWC measures and have achieved remarkable results [73,74,75,76].

As the KB is shared by four countries, there is uncertainty in the consolidation standards of road density and stream network. Different countries have different road construction standards, so the only way to choose whether the road is paved or not is to merge the road standard, which will weaken the spatial heterogeneity of the HII. A similar problem exists with stream network assignments. People using at least basic drinking water services make up 49.10%, 57.71%, 56.73%, and 60.83% of the population of Uganda, Rwanda, Tanzania, and Burundi, respectively [77]. Thus, the various populations have different levels of demand for the rivers, but we cannot identify the differences; hence, we need to assign the same criteria.

Furthermore, only precipitation factors that directly affect soil erosion were selected as the metrics for climate change. Meteorological factors that indirectly affect soil erosion (temperature, humidity et al.) must also be considered as part of future studies. Finally, more evidence concerning the SWCs [78], and agricultural measures [79] at the local scale would again improve upon the accuracy of the analysis, owing to the large extent of the KB.

## 5. Conclusions

The analysis of the impact of climate change and human activities on soil erosion is currently mostly concentrated on the impact of independent factors. This study adopted a grey relation analysis to conduct a qualitative analysis of the impact on soil erosion by climate change and human influence from a large-scale perspective. Taking the Kagera Basin in East Africa as an example, we conducted an innovative analysis of soil erosion problems under high population pressure and rapid economic development.

Our studies have shown that the basin experiences a very low level (10.54 t ha^−1^ yr^−1^) of soil erosion, dominated by rangeland and farmland areas, followed by forest, wetlands, and built-up areas. Climate change shows a more severe situation, with precipitation changing a great deal over the past 30 years, where the maximum value of Sen’s slope is 13.13 and the minimum value is −8.95. Meanwhile, the human influence intensity score is high at 21.93 (the maximum is 32). Through the evaluation of these three factors (climate change, human influence, and soil erosion), it is found that in the Kagera basin, climate change has a greater impact on soil erosion, with it having a greater impact over 90.32% of the area, while the area where the impact of human influence is the greater covers 9.68% of the basin.

## Figures and Tables

**Table 1:** International Geosphere-Biosphere Programme (IGBP) land use/land cover (LULC) types and the human influence scores of each type.

No.	LULC	Descriptions	Score
1	Evergreen broadleaf forests	Lands dominated by broadleaf woody vegetation with a percent cover > 60% and height exceeding 2 m.	0
2	Closed shrub-land	Lands with woody vegetation less than 2 m tall and with shrub canopy cover > 60%.	0
3	Open shrub-land	Lands with woody vegetation less than 2 m tall and with shrub canopy cover between 10 and 60%.	1
4	Woody savannas	Lands with herbaceous and other understory systems, and with forest canopy cover between 30 and 60%. The forest cover height exceeds 2 m.	1
5	Savannas	Lands with herbaceous and other understory systems, and with forest canopy cover between 10 and 30%. The forest cover height exceeds 2 m.	1
6	Grasslands	Lands with herbaceous types of cover. Tree and shrub cover together are less than 10%.	2
7	Permanent wetlands	Lands with a permanent mixture of water and herbaceous or woody vegetation.	1
8	Croplands	Lands covered with temporary crops followed by harvest and a bare soil period.	8
9	Urban and built-up lands	Land covered by buildings and other man-made structures.	10
10	Cropland/natural vegetation mosaic	Lands with a mosaic of croplands, forests, shrub-land, and grasslands in which no individual component comprises more than 60% of the landscape.	6
11	Water bodies	Lakes, reservoirs, and rivers. Can be either fresh or saltwater bodies.	0

**Table 2:** Human influence scores for roads and waterways.

Type		0–1 km	1–5 km	5–10 km	10–15 km
Roads	Paved	10	8	7	4
	Unpaved	6	4	2	1
Waterways		5	2		

**Table 3:** Soil erosion severity classes of the KB.

	Classes of the Soil Erosion					
Severity Classes	Very Low	Low	Moderate	High	Very High	Severe
Soil loss (t ha^−1^ yr^−1^)	0–5	5–10	10–25	25–50	50–100	>100
Area (%)	75.14	7.42	2.12	8.16	4.41	2.75

**Table 4:** The reclassification of LULC type according to the IGBP classification system.

LULC Type after Reclassification	Original IGBP LULC Type
Forest	Evergreen broadleaf forests
Rangeland	Closed shrub-land
	Open shrub-land
	Woody savannas
	Savannas
	Grasslands
Wetland	Permanent wetlands
Farmland	Croplands
	Cropland/natural vegetation mosaic
Built-up	Urban and built-up lands
Water	Water bodies

**Table 5:** Soil erosion of the different LULC types for the KB.

LULC Types	Soil Erosion Rate (t ha^−1^ yr^−1^)	Soil Loss (%)	Area	
			(%)	(km^2^)
Forest	0.71	0.08	1.22	698.25
Rangeland	8.51	49.60	61.35	35,071.75
Wetland	11.77	1.87	1.67	957.50
Farmland	13.61	45.97	35.56	20,330.50
Built-up	132.43	2.48	0.20	112.75

## Data Availability

Precipitation data used in this study are available from Climate Hazards Center UC Santa Barbara (https://data.chc.ucsb.edu/products/CHIRPS-2.0/, accessed on 20 October 2020). Soil data are available from the Africa Soil Information Service (http://africasoils.net/, accessed on 20 October 2020). The Digital Elevation Model, Normalized Difference Vegetation Index, and LULC data are available through the United States Geological Survey EarthExplorer database (https://earthexplorer.usgs.gov/, accessed on 20 October 2020). Population density data are available from Worldpop (https://www.worldpop.org/, accessed on 20 October 2020). Road data are available from the African Development Bank Group (https://www.afdb.org/, accessed on 20 October 2020). Stream networks data are available from the LakeVicFish Dataverse (https://dataverse.harvard.edu/dataverse/LakeVicFish, accessed on 20 October 2020). All data sources are public record.
